# Stage at diagnosis and breast cancer-specific mortality in breast cancer patients treated with antidepressants, anxiolytics, and antipsychotics: a population-based cohort study from Northern Ireland

**DOI:** 10.1007/s10549-025-07766-8

**Published:** 2025-07-05

**Authors:** Sarah M. Baxter, Charlene M. McShane, Stuart A. McIntosh, Damien Bennett, Lynne Lohfeld, Daniel R. S. Middleton, Gerard Savage, Deirdre Fitzpatrick, Joseph Kane, Ann McBrien, David McCallion, Anna Gavin, Chris R. Cardwell

**Affiliations:** 1 Centre for Public Health, Queen’s University Belfast, Belfast, Northern Ireland UK; 2 The Patrick G Johnston Centre for Cancer Research, Queen’s University Belfast, Belfast, Northern Ireland UK; 3 Breast Surgery Department, Belfast City Hospital, Belfast Health and Social Care Trust, Belfast, Northern Ireland UK; 4 Northern Ireland Cancer Registry, Belfast, UK; 5 Department of Psychiatry of Old Age, Belfast Health and Social Care Trust, Belfast, Northern Ireland UK; 6 Patient Representative, Northern, Ireland UK; 7 Patient Representative, England, UK

**Keywords:** Breast cancer, Mental health, Stage, Antidepressants, Antipsychotics

## Abstract

**Purpose:**

We examined the stage at diagnosis and breast cancer-specific mortality in a cohort of breast cancer patients prescribed medications used for mental health conditions before diagnosis.

**Methods:**

Women newly diagnosed with breast cancer from 2011 to 2021 were identified from the Northern Ireland Cancer Registry. The primary outcome was time to breast cancer-specific mortality up to March 2023. The secondary outcomes included stage at diagnosis. We identified anxiolytic, antidepressant, and antipsychotic prescriptions dispensed in the year before breast cancer diagnosis from the Northern Ireland Enhanced Prescribing Database. Cox regression models were used to calculate adjusted hazard ratios (aHR) and 95% confidence intervals (95%CIs) for cancer-specific mortality by use of medications.

**Results:**

We included 13,846 women with breast cancer. In the year before breast cancer diagnosis, 31.5% were dispensed antidepressants, 12.7% anxiolytics, and 3.5% antipsychotics. The odds of late-stage disease presentation in breast cancer patients dispensed medications for mental health conditions was similar to breast cancer patients not dispensed these medications, but patients dispensed antipsychotics had higher odds of unknown stage. We found no difference in the hazard rate of breast cancer-specific mortality in patients dispensed, versus not dispensed, anxiolytics (aHR = 1.06 95%CI 0.93–1.20), a small increase in patients dispensed, versus not dispensed, antidepressants (aHR = 1.11 95%CI 1.01–1.23) and a moderate increase in patients dispensed, versus not dispensed, antipsychotics (aHR = 1.45 95%CI 1.17–1.81).

**Conclusions:**

Breast cancer patients dispensed medications for mental health conditions were not at higher odds of presenting with late-stage disease, but patients dispensed antidepressants, and especially antipsychotics, had worse breast cancer-specific mortality.

**Supplementary Information:**

The online version contains supplementary material available at 10.1007/s10549-025-07766-8.

## Introduction

Mental health conditions could impact breast cancer outcomes by various mechanisms. Individuals with mental health conditions may find it challenging to engage with services and their symptoms may be incorrectly viewed as a feature of their mental health (diagnostic overshadowing) [1]. Mental health conditions may also represent obstacles to patients’ adherence to treatment regimens [2, 3]. Consequently, individuals with breast cancer and mental health conditions could receive later diagnoses [4] and/or incomplete treatment [5] potentially leading to poorer outcomes.

Although there has been much research on breast cancer survival and depression and anxiety, most of it has focussed on depression and anxiety after breast cancer diagnosis [6]. The studies investigating pre-existing mental health conditions and breast cancer survival have reported mixed findings. Most studies focussed on pre-existing depression [7–11], with much less research on pre-existing anxiety [6]. Similarly, there has been limited research on psychotic disorders and breast cancer survival. Some studies [7, 12–14] have shown worse survival in patients with pre-existing severe mental health conditions.

In Northern Ireland (NI), the impact of mental health conditions on breast cancer outcomes is of particular concern because mental health conditions are common [15] and a previous study observed marked reductions in breast cancer screening in patients with poor mental health [16]. Also, a UK Breast Cancer Care report on inequalities [17] highlighted the need for further research on breast cancer patients with mental health conditions. Consequently, our primary goal was to compare the hazard rate of breast cancer-specific mortality in female breast cancer patients dispensed medications which can be used for mental health conditions before diagnosis compared to those who were not dispensed these medications before diagnosis. We also compared the odds of presenting at late-stage diagnosis in female breast cancer patients dispensed medications which can be used for mental health conditions before diagnosis compared to those who were not dispensed these medications.

## Methods

### Data sources

The Northern Ireland Cancer Registry (NICR) captures data on all patients diagnosed with cancer in NI including tumour site, date of diagnosis, tumour characteristics, cancer treatment and death registration, with high accuracy and estimated completeness of over 99% [18]. The NICR acquires notifications from pathology reports, hospital admissions and discharges, and death registrations from the General Registrar Office. These data sources are combined electronically and manually to ensure data are coded to international cancer registration standards [19].

The Northern Ireland Enhanced Prescribing Database (NIEPD) captures all NHS prescriptions dispensed by community pharmacists in NI. The NIEPD and NICR were linked on a unique patient identifier (NI Health and Care Number) by the Health and Social Care Northern Ireland Honest Broker Service. Ethical approval for the study was obtained from Bradford Leeds Research Ethics Committee (reference: 23/YH/0157).

### Cohort

A cohort was identified from NICR of all women newly diagnosed with breast cancer (ICD-10 code C50) between January 2011 and December 2021. Women with a previous diagnosis of any cancer (apart from non-melanoma skin cancer) were excluded.

### Outcome

The primary outcome was breast cancer-specific mortality based on an underlying cause of death of breast cancer (ICD-10 code C50). The secondary outcomes were early-stage at diagnosis (Stage 1 to 3) and late-stage at diagnosis (defined as Stage 4 at diagnosis, or Stage 4 and Stage unknown at diagnosis) and all-cause mortality.

### Exposure

The receipt of prescriptions for anxiolytics, antidepressants, and antipsychotics was used as a proxy measure of mental health status. Prescription data have been previously utilised in epidemiological research to examine mental health [20, 21]. The prescriptions were identified from dispensing records from the NIEPD. The British National Formulary (BNF) [22] classification was used to identify anxiolytics (Sect.  4.1.2), antidepressants (Sect.  4.3) and antipsychotics (Sect.  4.2, excluding levomepromazine 6 mg tablets, levomepromazine injections and haloperidol injections as these are used for palliative care). Mood stabilisers (such as lithium, valproate, carbamazepine, and lamotrigine) used in the treatment of bipolar disorder and refractory depression were not included as most are often also used as anticonvulsant therapies. See Supplementary Table 1 for included medications. In the primary analysis, medications of interest were included if they were dispensed in the 12 months before breast cancer diagnosis.

### Covariates

The NICR provided year of diagnosis, age, stage, grade and pathway to diagnosis. Surgery, chemotherapy and radiotherapy within the first year of diagnosis were also taken from cancer registry records. Use of endocrine treatments such as tamoxifen or aromatase inhibitors were determined from dispensed medications from NIEPD. Comorbidities were identified from hospital admissions data up to 5 years prior to breast cancer diagnosis. The following conditions from the Charlson Comorbidity Index were identified (based on an ICD 10 code as a cause of hospital admission using previously used code lists [23]): myocardial infarction, congestive heart disease, peripheral vascular disease, cerebrovascular disease, chronic pulmonary disease, dementia, liver disease, peptic ulcer disease, diabetes and chronic kidney disease. Deprivation was determined from the postcode of residence using the 2017 NI Multiple Deprivation Measure [24].

### Statistical analysis

The characteristics of patients with breast cancer were determined by receipt of prescriptions for anxiolytics, antidepressants and antipsychotics.

Logistic regression analysis was conducted with late-stage at diagnosis as the outcome (based upon Stage 4 versus Stage 1 to 3) and separately for anxiolytics, antidepressants and antipsychotics as the exposure. An adjusted analysis was conducted including age, year of diagnosis, deprivation level and Charlson comorbidities in the model. This analysis was repeated considering late-stage as Stage 4 or Stage unknown versus Stage 1 to 3.

Cox regression analysis was conducted with time to breast cancer-specific mortality as the outcome (censoring on death from other causes, date of emigration and date of complete mortality records 31st March 2023) and separately for anxiolytics, antidepressants, and antipsychotics as the exposure. Kaplan–Meier survivor functions were determined, smoothed using a lowess smoother (to ensure disclosure control) and plotted to show the mortality by medication use. An adjusted analysis was conducted including age, year of diagnosis, deprivation and Charlson comorbidities in the model. Stage and grade were excluded from the adjusted analysis as arguably these lie on the causal pathway; however, an additional adjusted analysis was conducted including stage and grade to explore their impact on the associations. We conducted two further analyses: (a) we defined current prescriptions holders as those whose last (most recent) prescription was within three months of their cancer diagnosis and former prescription holders if their last (most recent) medication was between 3 and 12 months before their breast cancer diagnosis [2]; (b) we defined ‘new’ prescription holders as those who received their first (earliest) prescription within three months of their cancer diagnosis and ‘existing’ prescription holders if they received their first (earliest) prescription between three to twelve months before their breast cancer diagnosis.

We conducted various additional analyses and sensitivity analyses: (i) restricted to breast cancer patients under 70 years old to reduce the number of individuals receiving medications for symptoms of dementia; (ii) excluded prescriptions in the 3 months before cancer diagnosis, to exclude individuals who may have received prescriptions for early symptoms; (iii) for similar reasons, the main analysis was repeated only based upon prescriptions in the period 24–12 months before diagnosis; (iv) included prescriptions for all mental health conditions (i.e. anxiolytics, antidepressants and antipsychotics) in the model; (v) adjusted for stage and grade using multiple imputation with chained equations to impute missing stage and grade. Stage and grade were imputed in 10 datasets using ordinal logistic regression models with cancer-specific death status, cumulative hazard along with all confounders from the adjusted model included in imputation models [25], and results were combined using Rubin’s rules [26]; (vi) examined non-breast cancer-specific mortality (censoring on death from breast cancer) and all-cause mortality as outcomes; (vii) excluded individuals who died on their diagnosis date to investigate the impact of death certificate only diagnoses; (viii) compared individuals taking selective serotonin reuptake inhibitors (SSRIs) (as this class of antidepressants has fewer alternative indications) with individuals not taking antidepressants; (ix) compared individuals dispensed both antidepressants and anxiolytics (who may have more severe mental health conditions) with individuals not taking either; (x) compared individuals taking second generation antipsychotics (as this class of antipsychotics has fewer alternative indications) with individuals not taking antipsychotics. All analyses were conducted using STATA 18 (StataCorp, Texas, USA).

## Results

The cohort included 13,846 females with breast cancer. Overall, 12.7% were dispensed anxiolytics, 31.5% were dispensed antidepressants and 3.5% were dispensed antipsychotics in the year before diagnosis. Approximately 6.7% of women were dispensed antidepressants and anxiolytics, 1.3% were dispensed antidepressants and antipsychotics, and 1.1% of women were dispensed all three medications in the year before their breast cancer diagnosis. Please see Supplementary Fig. 1 for further information on the co-prescription of medications for mental health conditions.

### Characteristics of patients

The characteristics of patients who were dispensed anxiolytics, antidepressants and antipsychotics in the year before cancer diagnosis are shown in Table 1. Patients who were dispensed anxiolytics were similar to those who were not, apart from higher rates of antidepressant and antipsychotic prescriptions. Women who were dispensed antidepressants were generally similar to those who were not except they were from more deprived areas and had higher rates of anxiolytic and antipsychotic prescriptions. Women who were dispensed antipsychotics, compared to those were not, were older, from more deprived areas, had much lower breast cancer treatment rates (surgery, radiotherapy and chemotherapy) and had higher rates of prescriptions for anxiolytics and antidepressants.

**Table 1:** Characteristics of breast cancer patients by prescriptions for mental health conditions in year before diagnosis

Characteristic	Anxiolytic	No anxiolytic	Antidepressant	No antidepressant	Antipsychotic	No antipsychotic
*n*	1758	12,088	4358	9488	483	13,363
Year: 2010–13	502 (29%)	3076 (25%)	981 (23%)	2597 (27%)	122 (25%)	3456 (26%)
2014–16	500 (28%)	3249 (27%)	1193 (27%)	2556 (27%)	127 (26%)	3622 (27%)
2017–19	478 (27%)	3438 (28%)	1310 (30%)	2606 (27%)	141 (29%)	3775 (28%)
2020–22	278 (16%)	2325 (19%)	874 (20%)	1729 (18%)	93 (19%)	2510 (19%)
Age: < 50	271 (15%)	2588 (21%)	769 (18%)	2090 (22%)	53 (11%)	2806 (21%)
50– < 60	396 (23%)	3037 (25%)	1207 (28%)	2226 (23%)	121 (25%)	3312 (25%)
60– < 70	420 (24%)	2962 (25%)	1107 (25%)	2275 (24%)	122 (25%)	3260 (24%)
70– < 80	385 (22%)	1981 (16%)	767 (18%)	1599 (17%)	88 (18%)	2278 (17%)
80 +	286 (16%)	1520 (13%)	508 (12%)	1298 (14%)	99 (20%)	1707 (13%)
Deprivation (in fifths):						
1st (most deprived)	355 (20%)	1955 (16%)	893 (20%)	1417 (15%)	132 (27%)	2178 (16%)
5th (least deprived)	315 (18%)	2668 (22%)	784 (18%)	2199 (23%)	73 (15%)	2910 (22%)
Stage: 1	686 (42%)	4778 (41%)	1747 (42%)	3717 (41%)	164 (40%)	5300 (41%)
2	635 (39%)	4677 (40%)	1668 (40%)	3644 (40%)	172 (42%)	5140 (40%)
3	217 (13%)	1487 (13%)	518 (12%)	1186 (13%)	49 (12%)	1655 (13%)
4	104 (6%)	615 (5%)	222 (5%)	497 (5%)	26 (6%)	693 (5%)
Missing	116	531	203	444	72	575
Grade: 1	192 (12%)	1373 (12%)	520 (13%)	1045 (12%)	57 (14%)	1508 (12%)
2	801 (49%)	5709 (50%)	2066 (50%)	4444 (50%)	201 (48%)	6309 (50%)
3	648 (39%)	4401 (38%)	1563 (38%)	3486 (39%)	159 (38%)	4890 (38%)
Missing	117	605	209	513	66	656
Pathway: screen detected	450 (26%)	3562 (29%)	1278 (29%)	2734 (29%)	133 (28%)	3879 (29%)
Red flag referral	931 (53%)	6223 (51%)	2281 (52%)	4873 (51%)	241 (50%)	6913 (52%)
Death cert only	0–10^a^	30–40^a^	16 (0%)	24 (0%)	12 (2%)	28 (0%)
Other	367–377^a^	2263–2273^a^	783 (18%)	1857 (20%)	97 (20%)	2543 (19%)
Treatment in year after diagnosis:						
Mastectomy	472 (27%)	3141 (26%)	1131 (26%)	2482 (26%)	105 (22%)	3508 (26%)
Breast-conserving	862 (49%)	6494 (54%)	2337 (54%)	5019 (53%)	201 (42%)	7155 (54%)
Radiotherapy	1127 (64%)	8660 (72%)	3050 (70%)	6737 (71%)	259 (54%)	9528 (71%)
Chemotherapy	518 (29%)	4527 (37%)	1516 (35%)	3529 (37%)	119 (25%)	4926 (37%)
Tamoxifen^b^	575 (33%)	4684 (39%)	1500 (34%)	3759 (40%)	117 (24%)	4714 (35%)
Aromatase inhibitors^b^	1198 (68%)	7693 (64%)	2906 (67%)	5985 (63%)	291 (60%)	6497 (49%)
Prescriptions in year before diagnosis:						
Anxiolytic			1080 (25%)	678 (7%)	188 (39%)	1570 (12%)
Antidepressants	1080 (61%)	3278 (27%)			343 (71%)	4015 (30%)
Antipsychotic	188 (11%)	295 (2%)	343 (8%)	140 (1%)		

^a^Range given to avoid disclosure of small counts

^b^Measured at any time after diagnosis

### Late-stage at diagnosis

The association between prescriptions for mental health conditions and late-stage presentation at diagnosis is shown in Table 2. Overall, in breast cancer patients, there was limited evidence of a difference in the odds of patients diagnosed at late-stage who were dispensed anxiolytics compared with those who were not (6.3% vs. 5.3% Stage 4; aOR = 1.11 95% CI 0.89–1.38). In breast cancer patients, the odds of patients diagnosed at late-stage was similar for those dispensed antidepressants compared with those who were not (5.3% vs 5.5% Stage 4; aOR = 0.94 95% CI 0.80–1.11) and in those dispensed antipsychotics compared to those who were not (6.3% versus 5.4% Stage 4; aOR = 1.11 95% CI 0.74–1.68). The results were similar when late-stage was expanded to include Stage unknown and Stage 4. However, patients dispensed antipsychotics had higher odds of late-stage diagnosis compared with patients not dispensed antipsychotics (20.3% vs 9.5% Stage 4/Stage unknown; aOR = 2.14 95% CI 1.68–2.73).

**Table 2:** Association between prescriptions for mental health conditions in the year before diagnosis and late-stage at diagnosis

Prescriptions in year prior to diagnosis	Late-stage % (*n*/*N*)	Unadjusted HR (95% CI)	*P*	Adjusted^a^ HR (95% CI)	*P*
Late-stage (stage 4) versus early-stage (stage 1 to 3)					
Anxiolytic					
None	5.3% (615/11557)	1.00 (Ref Cat.)		1.00 (Ref Cat.)	
1 or more	6.3% (104/1642)	1.20 (0.97, 1.49)	0.091	1.11 (0.89, 1.38)	0.362
Antidepressant					
None	5.5% (497/9044)	1.00 (Ref Cat.)		1.00 (Ref Cat.)	
1 or more	5.3% (222/4155)	0.97 (0.82, 1.14)	0.72	0.94 (0.80, 1.11)	0.48
Antipsychotic					
None	5.4% (693/12788)	1.00 (Ref. Cat.)		1.00 (Ref. Cat.)	
1 or more	6.3% (26/411)	1.18 (0.79, 1.77)	0.426	1.11 (0.74, 1.68)	0.612
Late-stage (stage 4 or missing) versus early-stage (stage 1 to 3)					
Anxiolytic					
None	9.5% (1146/12088)	1.00 (Ref. Cat.)		1.00 (Ref. Cat.)	
1 or more	12.5% (220/1758)	1.37 (1.17, 1.59)	< 0.001	1.15 (0.98, 1.35)	0.095
Antidepressant					
None	9.9% (941/9488)	1.00 (Ref. Cat.)		1.00 (Ref. Cat.)	
1 or more	9.8% (425/4358)	0.98 (0.87, 1.11)	0.762	1.02 (0.89, 1.15)	0.815
Antipsychotic					
None	9.5% (1268/13363)	1.00 (Ref. Cat.)		1.00 (Ref. Cat.)	
1 or more	20.3% (98/483)	2.43 (1.93, 3.05)	< 0.001	2.14 (1.68, 2.73)	< 0.001

^a^Model contains age (in years), year of diagnosis (in years), deprivation, comorbidities (myocardial infarction, congestive heart disease, peripheral vascular disease, cerebrovascular disease, chronic pulmonary disease, dementia, liver disease, peptic ulcer disease, diabetes and chronic kidney disease)

### Treatment for mental health conditions and breast cancer outcomes

**Fig. 1:**
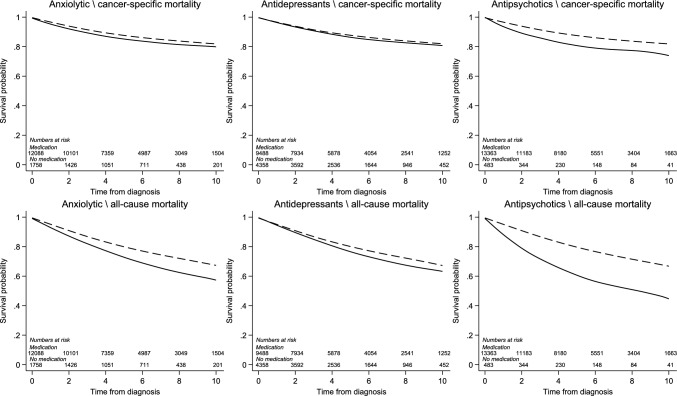
Smoothed Kaplan–Meier survival curves for breast cancer-specific and all-cause mortality in prescription holders (solid line) and non-holders (dashed line)

The association between prescriptions for anxiolytics, antidepressants and antipsychotics in the year before breast cancer diagnosis and breast cancer-specific mortality are shown in Table 3 (main analysis), Table 4 (sensitivity and secondary analysis) and Fig. 1. The comparison in each analysis was breast cancer patients who were not dispensed the medication of interest.

**Table 3:** Association between prescriptions for mental health conditions and breast cancer-specific mortality

Condition	Nos	Cancer-specific deaths	Person years	Unadjusted HR (95% CI)	*P*	Adjusted^a^ HR (95% CI)	*P*	Adjusted (+ stage/grade)^b^ HR (95% CI)	*P*
Anxiolytics									
Prescription in year before diagnosis									
No anxiolytic	12,088	1554	66,364	1.00 (Ref. Cat.)		1.00 (Ref. Cat.)		1.00 (Ref. Cat.)	
1 or more anxiolytic	1758	266	9453	1.20 (1.05, 1.37)	0.006	1.06 (0.93, 1.20)	0.411	0.95 (0.82, 1.11)	0.539
Most recent prescription in year prior									
0–3 months before diagnosis (current holder)	1013	163	5215	1.32 (1.12, 1.55)	0.001	1.13 (0.96, 1.33)	0.151	1.05 (0.87, 1.26)	0.626
> 3 months before diagnosis (former holder)	745	103	4238	1.05 (0.86, 1.28)	0.646	0.96 (0.79, 1.18)	0.709	0.84 (0.66, 1.05)	0.128
Time initiating treatment in year prior									
0–3 months before diagnosis (new holder)	218	43	1157	1.59 (1.18, 2.16)	0.003	1.50 (1.10, 2.03)	0.01	1.24 (0.87, 1.76)	0.235
> 3 months before diagnosis (existing holder)	1540	223	8296	1.15 (1.00, 1.32)	0.058	1.00 (0.87, 1.15)	0.996	0.91 (0.78, 1.07)	0.274
Antidepressants									
Prescription in year before diagnosis									
No antidepressant	9488	1228	53,027	1.00 (Ref. Cat.)		1.00 (Ref. Cat.)		1.00 (Ref. Cat.)	
1 or more antidepressant	4358	592	22,790	1.10 (0.99, 1.21)	0.066	1.11 (1.00, 1.22)	0.042	1.16 (1.04, 1.30)	0.009
Most recent prescription in year prior									
0–3 months before diagnosis (current holder)	3366	459	17,275	1.12 (1.00, 1.24)	0.041	1.13 (1.01, 1.26)	0.028	1.20 (1.06, 1.35)	0.004
> 3 months before diagnosis (former holder)	992	133	5515	1.03 (0.86, 1.23)	0.767	1.04 (0.87, 1.25)	0.66	1.04 (0.85, 1.28)	0.681
Time initiating treatment in year prior									
0–3 months before diagnosis (new holder)	192	32	1068	1.30 (0.91, 1.85)	0.144	1.31 (0.92, 1.86)	0.132	1.19 (0.80, 1.77)	0.4
> 3 months before diagnosis (existing holder)	4166	560	21,721	1.09 (0.98, 1.20)	0.103	1.10 (0.99, 1.21)	0.069	1.16 (1.03, 1.30)	0.011
Antipsychotics									
Prescription in year before diagnosis									
No antipsychotic	13,363	1734	73,637	1.00 (Ref Cat.)		1.00 (Ref Cat.)		1.00 (Ref. Cat.)	
1 or more antipsychotic	483	86	2180	1.61 (1.30, 2.00)	< 0.001	1.45 (1.17, 1.81)	0.001	1.47 (1.13, 1.92)	0.005
Most recent prescription in year prior									
0–3 months before diagnosis (current holder)	377	70	1690	1.69 (1.33, 2.15)	< 0.001	1.54 (1.21, 1.96)	< 0.001	1.48 (1.11, 1.98)	0.008
> 3 months before diagnosis (former holder)	106	16	489	1.33 (0.81, 2.18)	0.255	1.17 (0.71, 1.92)	0.54	1.40 (0.73, 2.70)	0.314
Time initiating treatment in year prior									
0–3 months before diagnosis (new holder)	18	0–10^c^	69	5.23 (2.72, 10.1)	< 0.001	4.34 (2.25, 8.38)	< 0.001	2.95 (1.53, 5.71)	0.001
> 3 months before diagnosis (existing holder)	465	76–86^c^	2110	1.49 (1.19, 1.87)	0.001	1.35 (1.07, 1.70)	0.011	1.34 (1.00, 1.79)	0.047

^a^Model contains age (in years), year of diagnosis (in years), deprivation, comorbidities (myocardial infarction, congestive heart disease, peripheral vascular disease, cerebrovascular disease, chronic pulmonary disease, dementia, liver disease, peptic ulcer disease, diabetes and chronic kidney disease)

^b^Model same as ^a^ plus stage and grade using complete case analysis

^c ^Range given to avoid disclosure of small counts

**Table 4:** Sensitivity and secondary analyses for association between prescriptions for mental health conditions and breast cancer-specific mortality

Sensitivity analysis	Nos	Events	Person years	Unadjusted HR (95% CI)	*P*	Adjusted^a^ HR (95% CI)	*P*	Adjusted (+ stage/grade)^b^ HR (95% CI)	*P*
Anxiolytics^c^									
Main analysis	13,846	1820	75,817	1.20 (1.05, 1.37)	0.006	1.06 (0.93, 1.20)	0.411	0.95 (0.82, 1.11)	0.539
Under 70 years old	9674	885	57,679	1.10 (0.90, 1.35)	0.338	1.07 (0.87, 1.31)	0.513	0.93 (0.75, 1.16)	0.534
Excluding prescriptions 3 months before diagnosis^d^	13,846	1820	75,817	1.13 (0.99, 1.30)	0.079	0.99 (0.86, 1.14)	0.897	0.91 (0.77, 1.07)	0.248
Using prescriptions 24 to 12 months before diagnosis^e^	12,654	1580	65,248	1.13 (0.97, 1.31)	0.119	1.01 (0.87, 1.17)	0.885	1.01 (0.85, 1.20)	0.885
Also adjusting for antidepressants and antipsychotics^f^	13,846	1820	75,817	1.20 (1.05, 1.37)	0.006	1.00 (0.88, 1.15)	0.955	0.89 (0.77, 1.05)	0.162
Adjusting for stage and grade using multiple imputation	13,846	1820	75,817	1.20 (1.05, 1.37)	0.006	1.06 (0.93, 1.20)	0.411	1.03 (0.89, 1.19)	0.697
Excluding patients who die on diagnosis date	13,802	1795	75,817	1.20 (1.05, 1.37)	0.007	1.06 (0.93, 1.21)	0.409	0.95 (0.82, 1.11)	0.539
Non breast cancer-specific mortality	13,846	1557	75,817	1.74 (1.53, 1.97)	< 0.001	1.43 (1.26, 1.62)	< 0.001	1.51 (1.31, 1.74)	< 0.001
Outcome all-cause mortality	13,846	3377	75,817	1.44 (1.32, 1.57)	< 0.001	1.22 (1.11, 1.33)	< 0.001	1.20 (1.08, 1.32)	0.001
Antidepressants^g^									
Main analysis	13,846	1820	75,817	1.10 (0.99, 1.21)	0.066	1.11 (1.00, 1.22)	0.042	1.16 (1.04, 1.30)	0.009
SSRIs^h^	12,229	1584	67,557	1.04 (0.92, 1.17)	0.534	1.09 (0.97, 1.23)	0.15	1.11 (0.97, 1.27)	0.121
Antidepressant plus anxiolytic^i^	9890	1252	55,064	1.11 (0.93, 1.32)	0.249	1.03 (0.86, 1.23)	0.741	1.05 (0.86, 1.28)	0.626
Under 70 years old	9674	885	57,679	1.08 (0.94, 1.25)	0.26	1.07 (0.93, 1.24)	0.335	1.15 (0.99, 1.33)	0.074
Excluding prescriptions in 3 months before diagnosis^d^	13,846	1820	75,817	1.08 (0.98, 1.19)	0.128	1.09 (0.99, 1.21)	0.088	1.15 (1.03, 1.29)	0.013
Using prescriptions 24 to 12 months before diagnosis^e^	12,654	1580	65,248	1.05 (0.95, 1.17)	0.339	1.07 (0.96, 1.19)	0.224	1.17 (1.04, 1.32)	0.01
Also adjusting for anxiolytics and antipsychotics^f^	13,846	1820	75,817	1.10 (0.99, 1.21)	0.066	1.08 (0.98, 1.20)	0.135	1.16 (1.03, 1.30)	0.011
Adjusting for stage and grade using multiple imputation	13,846	1820	75,817	1.10 (0.99, 1.21)	0.066	1.11 (1.00, 1.22)	0.042	1.17 (1.05, 1.29)	0.003
Excluding patients who die on diagnosis date	13,802	1795	75,817	1.09 (0.99, 1.20)	0.09	1.10 (1.00, 1.22)	0.06	1.16 (1.04, 1.30)	0.009
Non breast cancer-specific mortality	13,846	1557	75,817	1.29 (1.17, 1.43)	< 0.001	1.42 (1.28, 1.58)	< 0.001	1.48 (1.32, 1.66)	< 0.001
Outcome all-cause mortality	13,846	3377	75,817	1.18 (1.10, 1.27)	< 0.001	1.23 (1.14, 1.32)	< 0.001	1.27 (1.17, 1.38)	< 0.001
Antipsychotics^j^									
Main analysis	13,846	1820	75,817	1.61 (1.30, 2.00)	< 0.001	1.45 (1.17, 1.81)	0.001	1.47 (1.13, 1.92)	0.005
Second generation antipsychotics^k^	13,742	1798	75,278	1.58 (1.23, 2.02)	< 0.001	1.45 (1.13, 1.87)	0.003	1.35 (0.98, 1.85)	0.065
Under 70 years old	9674	885	57,679	1.60 (1.16, 2.21)	0.004	1.55 (1.12, 2.15)	0.008	1.54 (1.08, 2.19)	0.016
Excluding prescriptions in 3 months before diagnosis^d^	13,846	1820	75,817	1.48 (1.18, 1.86)	0.001	1.34 (1.07, 1.69)	0.012	1.34 (1.00, 1.79)	0.05
Using prescriptions 24 to 12 months before diagnosis^e^	12,654	1580	65,248	1.50 (1.17, 1.92)	0.001	1.41 (1.10, 1.81)	0.007	1.17 (0.84, 1.62)	0.346
Also adjusting for anxiolytics and antidepressants^f^	13,846	1820	75,817	1.61 (1.30, 2.00)	< 0.001	1.41 (1.13, 1.76)	0.002	1.42 (1.08, 1.86)	0.011
Adjusting for stage and grade using multiple imputation	13,846	1820	75,817	1.61 (1.30, 2.00)	< 0.001	1.45 (1.17, 1.81)	0.001	1.50 (1.17, 1.93)	0.002
Excluding patients who die on diagnosis date	13,802	1795	75,817	1.52 (1.21, 1.90)	< 0.001	1.37 (1.10, 1.72)	0.006	1.47 (1.13, 1.92)	0.005
Non breast cancer-specific mortality	13,846	1557	75,817	2.85 (2.37, 3.44)	< 0.001	2.40 (1.98, 2.90)	< 0.001	2.36 (1.87, 2.99)	< 0.001
Outcome all-cause mortality	13,846	3377	75,817	2.16 (1.88, 2.49)	< 0.001	1.87 (1.62, 2.16)	< 0.001	1.86 (1.56, 2.21)	< 0.001

^a^Model contains age (in years), year of diagnosis (in years), deprivation, comorbidities (myocardial infarction, congestive heart disease, peripheral vascular disease, cerebrovascular disease, chronic pulmonary disease, dementia, liver disease, peptic ulcer disease, diabetes and chronic kidney disease)

^b^Model same as ^a^ plus stage and grade

^c^Anxiolytics users vs non-users

^d^Prescribing based upon 12 months to 3 months before breast cancer diagnosis

^e^Prescribing based upon 24 to 12 months before diagnosis excluding those diagnosed in 2011 (as do not have full records)

^f^Models ^a^ and ^b^ additionally include prescriptions for anxiolytics, antidepressants and antipsychotics in year prior diagnosis

^g^Antidepressants users vs non-users

^h^Comparing SSRI prescription holders to non-prescription holders of antidepressants

^i^Comparing prescription holders of both antidepressants and anxiolytics to prescription holders of neither

^j^Antipsychotics users vs non-users

^k^Comparing second generation antipsychotic users to non-users of antipsychotics

We found no evidence of an increase in hazard rate of breast cancer-specific mortality in those dispensed anxiolytics after adjusting for age, year, deprivation, and comorbidities (aHR 1.06 95% CI 0.93–1.20), and after additionally adjusting for stage and grade (aHR 0.95 95% CI 0.82–1.11). The lack of association was similar in analysis of current and new prescription holders. The findings were generally similar in most sensitivity and secondary analyses. There was a small increase the hazard rate of all-cause mortality (aHR 1.20 95% CI 1.08–1.32) which largely reflected non breast cancer-specific mortality (aHR 1.51 95% CI 1.31–1.74) (see Table 4).

There was a small increase in the hazard rate of breast cancer-specific mortality in patients dispensed antidepressants after adjusting for age, year, deprivation and comorbidities (aHR 1.11 95% CI 1.00–1.22). This association remained after additional adjustment for stage and grade of breast cancer (aHR 1.16 95% CI 1.04–1.30). The association was similar in current and new prescription holders and in most sensitivity analyses. There was an increase in the hazard rate of all-cause mortality (aHR 1.27 95% CI 1.17–1.38) which was stronger for non-breast cancer-specific mortality (aHR 1.48 95% CI 1.32–1.66) (see Table 4).

There was an increase in the hazard rate of breast cancer-specific mortality in breast cancer patients dispensed antipsychotics after adjustment for age, year, deprivation and comorbidities (aHR 1.45 95% CI 1.17–1.81), which was not attenuated after additional adjustment for stage and grade (aHR 1.47 95% CI 1.13–1.92). The association was apparent in current prescription holders (aHR 1.48 95% CI 1.11–1.98) and marked in patients who were new prescription holders (aHR 2.95 95% CI 1.53–5.71). The associations were similar in sensitivity analysis for instance when excluding prescriptions in the 3 months before diagnosis (aHR 1.34 95% CI 1.07–1.69). There was a marked increase in the hazard rate of all-cause mortality in patients dispensed antipsychotics (aHR 1.86 95% CI 1.56–2.21) which was particularly strong for non-breast cancer-specific mortality (aHR 2.36 95% CI 1.87, 2.99) (see Table 4).

## Discussion

In this population-based study of almost 14,000 breast cancer patients, we found little difference in the odds of late-stage disease presentation (Stage 4) in those dispensed anxiolytics, antidepressants and antipsychotics compared to those who were not dispensed these medications. However, women dispensed antipsychotics were more likely to have an unknown stage at diagnosis. Furthermore, we observed an 11% increase in the hazard rate of early breast cancer-specific mortality in patients who were dispensed antidepressants and a 45% increase in the hazard rate of early breast cancer-specific mortality in patients who were dispensed antipsychotics. The associations largely remained after adjustment for stage and grade. This suggests that late diagnosis does not explain the poorer mortality outcomes we found for patients dispensed antidepressants or antipsychotics.

Our findings for antipsychotics are generally consistent with previous studies that have shown poorer breast cancer-specific mortality in breast cancer patients with severe mental health conditions before diagnosis. A study from New Zealand showed a 65% increase in breast cancer-specific mortality in patients with schizophrenia and bipolar disorder [27]. A Finnish study reported a 50% increase in cancer-specific mortality in breast cancer patients with psychosis [12]. Two US studies [7, 28] also observed increases in breast cancer-specific mortality of around 20% in patients with severe mental health conditions, although only the latter study found this increase to be statistically significant [28]. Similarly, a Danish study found a 30% increase in breast cancer-specific mortality in women with a severe mental health condition [13].

Our findings for antidepressants and anxiolytics are consistent with previous studies which have shown weak associations with depression and anxiety and poorer breast cancer-specific mortality in women with breast cancer. For instance, a US cohort study showed no increase in breast cancer-specific mortality in women with depression or anxiety before diagnosis [7]. A UK study showed a 23% increase in all-cause mortality in women with depression before diagnosis [8]. A large US study showed an increase in all-cause and some evidence of an increase in breast cancer-specific mortality with depression before diagnosis particularly with newly developed depression before diagnosis [9]. Another US study showed an association between pre-diagnosis depression and survival [10]. A Swedish study showed an 11% increase in breast cancer-specific mortality in breast patients given antidepressants before diagnosis and suggested this could reflect treatment which deviated from guideline [11]. A Danish study observed an increase in mortality particularly in patients initiating treatment in the four-month period immediately before breast cancer diagnosis [2].

The cause of poorer outcomes in breast cancer patients who were dispensed medications for mental health conditions before diagnosis is unknown. Previous studies have suggested individuals with mental health conditions may have later stage at diagnosis as they may take longer to access help, their mental health condition may mask their physical symptoms, healthcare professionals may focus on their mental health rather than exploring emerging symptoms and they may face stigma from healthcare professionals [1, 2, 29, 30]. We observed little evidence of increased odds of late-stage disease presentation in patients who were dispensed medications for mental health conditions, and associations with breast cancer-specific mortality persisted after adjustment for stage. However, patients dispensed antipsychotics were more likely to have unknown stage which could reflect less complete diagnostic procedures and reduced levels of surgery in these patients who may have more severe mental illness. A previous US study also found a higher proportion cancer patients with psychosis had unknown stage [31], although a UK study found no association between psychiatric diagnosis and unknown stage, except for dementia [32].

Another possible reason for the observed poorer breast cancer outcomes in women who were dispensed medication for mental health conditions may be physical health disparities, particularly in those with severe mental health conditions. Prior research in NI has shown that patients with mental health conditions had a greater likelihood of multiple chronic conditions and all-cause mortality [33]. Further, a US study observed a 49% increase in overall mortality among breast cancer patients using antipsychotics [14]. In our study, we observed increased all-cause mortality across all three classes of medication used to treat mental health conditions in breast cancer patients, with an 80% increase in those dispensed antipsychotics (aHR 1.80, 95% CI 1.57–2.07). Although we adjusted for comorbidities, these may be underestimated because as we only had access to hospital diagnoses and therefore residual confounding is possible.

Other studies have suggested that worse cancer outcomes in patients with mental health conditions could reflect poorer treatment adherence or less comprehensive treatment. Several barriers to breast cancer screening for those with severe mental health conditions have been reported, including poor communication, stigmatising attitudes, and negative experiences, which can lead to mistrust and discourage attendance at future appointments [34, 35]. Similar barriers in cancer trajectories have been reported, such as communication challenges, low adherence to treatment regimens, stigma and fragmentation of care [3]. Furthermore, some studies have shown reductions in rates of definitive surgery and fewer chemotherapy and radiotherapy sessions in patients with mental illness [36–38], which would negatively impact breast cancer-specific survival. Adherence to endocrine therapy is an important factor in breast cancer survival with non-adherence and non-persistence associated with poorer breast cancer survival [39]. Mental illness has previously been associated with a modest reduction in initiation, adherence and persistence of endocrine treatment, which consequently impacts breast cancer survival [40]. The impact of breast cancer treatments was not a focus of this study, and we had limited information on treatments but only patients on antipsychotics appeared to have lower rates of certain cancer treatments. Future research could further investigate the impact of mental health conditions on rates and adherence with cancer treatments in this population.

The main strength of our study is the population-based cohort of breast cancer patients and identification of mental health conditions using a dispensing database that captures all dispensed NHS prescriptions in NI where prescriptions were free of charge during the study period. None of the medications investigated were available over the counter during the study period reducing misclassification.

However, our study had some limitations. The medications investigated are sometimes prescribed for indications other than mental health. However, the studies have shown a large proportion of users of antipsychotics (particularly second-generation antipsychotics) have diagnoses of psychosis, bipolar disorder or depression [41] and many users of antidepressants (particularly SSRIs) have diagnoses of depression or anxiety [42, 43]. Reassuringly, our results were similar when we investigated specifically second-generation antipsychotics or SSRIs. Further, a previous Northern Irish study using the same prescribing dataset showed prescriptions for psychotropic medications were strongly associated with both self-reported mental health and suicide risk [44]. Our exposure definition was based upon presumed mental health conditions and therefore our results may not apply to undiagnosed or misdiagnosed mental health conditions or those treated using non-pharmacological methods such as cognitive behavioural therapies. Furthermore, within types of mental health conditions we considered, there may be potential for misclassification as we are basing our definition on dispensed medications rather than diagnoses. For example, SSRIs can be used to treat both anxiety and depression, and antipsychotics can be combined with antidepressants as a means of augmenting antidepressants in the absence of psychosis [45, 46]. However, these classes may be considered a proxy for severity or refractoriness of mental health conditions, with the antipsychotics representing the most severe mental health conditions. Additionally, we were unable to adjust for potential confounders (e.g. smoking and alcohol use) that are associated with both mental health conditions and breast cancer [47–50]. We were not able to investigate cancer recurrence directly in our study. As we did not have access to a cancer-free control group we could not investigate aetiological associations between medications used to treat mental health conditions and risk of early versus late-stage breast cancer. Finally, it is possible that some deaths may have been incorrectly misclassified when determining cancer-specific mortality, but, as we are comparing groups and differential misclassification between groups seems unlikely, this weakness should have minimal impact on our observed associations [51].

In conclusion, in our breast cancer cohort we found no evidence of a difference in the odds of late-stage presentation at diagnosis in patients dispensed medications for mental health conditions compared with those who were not dispensed medications for mental health conditions. However, we found evidence that individuals dispensed antidepressants and particularly those dispensed antipsychotics, had an increased hazard rate of early breast cancer-specific mortality. Our findings will enable healthcare professionals to identify and focus on breast cancer patients most at risk of poorer outcomes and to tailor care for these individuals. As the cause of the poor outcomes is unknown, particularly in those who were dispensed antipsychotics, further research is warranted.

## Supplementary Information

Below is the link to the electronic supplementary material.

Supplementary file1 (DOCX 104 KB)

## Data Availability

Anonymised data can be accessed for authorised researchers, at a cost, after application to the Health and Social Care NI Honest Broker Service. Further information on how to apply can be found here: https://bso.hscni.net/directorates/digital/honest-broker-service/. Analysis codes are available upon request to the authors.
