# Supplementary material for: Stage at diagnosis and breast cancer-specific mortality in breast cancer patients treated with antidepressants, anxiolytics, and antipsychotics: a population-based cohort study from Northern Ireland
Source: Breast Cancer Res Treat. 2025 Jul 5;213(1):137–50. doi: 10.1007/s10549-025-07766-8 (PMC12259808; doi:10.1007/s10549-025-07766-8)
Supplement: Supplementary file 1 — Supplementary file1 (DOCX 104 KB) [file 10549_2025_7766_MOESM1_ESM.docx]

**Stage at diagnosis and breast cancer-specific mortality in breast cancer patients treated with antidepressants, anxiolytics, and antipsychotics: A population-based cohort study from Northern Ireland.**

**Supplementary Materials**

**Breast Cancer Treatment and Research**

Sarah M. Baxter, Charlene M. McShane, Stuart A McIntosh, Damien Bennett, Lynne Lohfeld, Daniel R.S. Middleton, Gerard Savage, Deidre Fitzpatrick, Joseph Kane, Ann Mc Brien, David McCallion, Anna Gavin, Chris R. Cardwell.

**Corresponding author**: Dr Sarah Baxter, Centre for Public Health, Queen’s University Belfast, Belfast, Northern Ireland, UK. Email: [S.Baxter@qub.ac.uk](mailto:S.Baxter@qub.ac.uk)

**Supplementary Table 1** Included medications

| **Anxiolytics** | **Antidepressants** | **Antipsychotics** |
| --- | --- | --- |
| Diazepam | Citalopram | Quetiapine |
| Buspirone | Amitriptyline | Risperidone |
| Lorazepam | Sertraline |  |
| Oxazepam | Fluoxetine | Olanzapine |
| Chlordiazepoxide | Venlafaxine | Chlorpromazine |
| Loprazolam | Mirtazapine |  |
|  | Escitalopram | Aripiprazole |
|  | Duloxetine | Haloperidol^a^ |
|  | Dosulepin | Amisulpride |
|  | Paroxetine | Paliperidone |
|  | Trazodone | Flupentixol decanoate |
|  | Clomipramine | Promazine |
|  | Nortriptyline | Trifluoperazine |
|  | Lofepramine | Sulpiride |
|  | Imipramine | Zuclopenthixol decanoate |
|  | Reboxetine | Haloperidol decanoate |
|  | Flupentixol dihydrochloride^b^ | Fluphenazine decanoate |
|  | Trimipramine | Zuclopenthixol |
|  | Agomelatine |  |
|  | Vortioxetine | Lurasidone |
|  | Fluvoxamine | Perphenazine |
|  | Doxepin | Flupentixol dihydrochloride^c^ ^c^ |
|  | Moclobemide | Levomepromazine^d^ |
|  | Tryptophan |  |
|  | Mianserin |  |
|  | Amitriptyline + Perphenazine |  |

^a^Excluding solution for injection.

^b^Flupentixol dihydrocodeine strength of 500mcg or 1mg.

^c^Flupentixol dihydrocodeine strength of 3mg.

^d^Exclusing solution for injection and 6mg tablets.

**Supplementary Figure 1** Co-prescribing of medications for mental health conditions in the year prior to breast cancer diagnosis


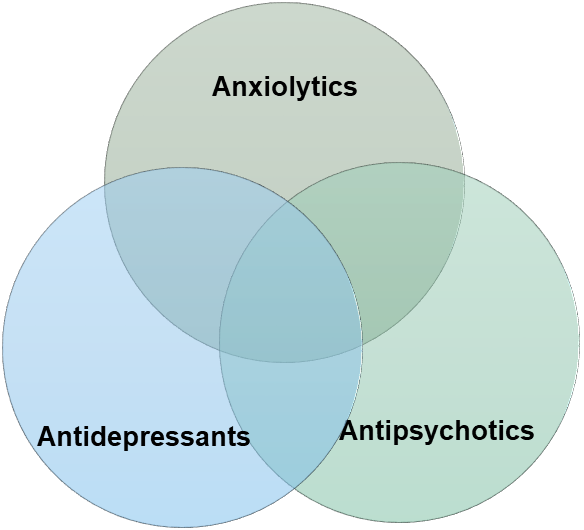


30 (0.2%)

110 (0.8%)

3,093 (22.3%)

922 (6.7%)

185 (1.3%)

**No medication for mental health conditions**

8,700 (62.8%)

158 (1.1%)

648 (4.7%)
